# Radiolabeled Angiopep‐2 Peptide Vector as a Preclinical Platform for Blood–Brain Barrier Targeting: Synthesis, Radiolabeling, and Preliminary In Vivo Biodistribution in Mice

**DOI:** 10.1002/psc.70109

**Published:** 2026-06-10

**Authors:** Evgenia Fotou, Adamantia Apostolopoulou, Christina‐Georgia Bika, Maria Giannakopoulou, Danai‐Efraimia Bajwa, Vassilios Moussis, Vassilios Tsikaris, Ioannis P. Gerothanassis, George A. Alexiou, Penelope Bouziotis, Andreas G. Tzakos

**Affiliations:** ^1^ Section of Organic Chemistry and Biochemistry, Department of Chemistry University of Ioannina Ioannina Greece; ^2^ Institute of Nuclear & Radiological Sciences & Technology, Energy & Safety National Center for Scientific Research “Demokritos” Athens Greece; ^3^ Neurosurgical Institute University of Ioannina Ioannina Greece

**Keywords:** ^161^Tb, ^177^Lu, Angiopep‐2, BBB, DOTA, glioma targeting, LRP1, peptide conjugates, radiolabeling

## Abstract

Brain tumor therapy remains limited by the blood–brain barrier (BBB), which restricts drug access. BBB‐penetrating peptides offer a promising strategy for delivering therapeutic and diagnostic payloads. Angiopep‐2 is a well‐established vector, yet novel radioconjugates based on this vector remain of interest. We report the synthesis and evaluation of DOTA‐Angiopep‐2 for radiolabeling with Lutetium‐177 (^177^Lu) and Terbium‐161 (^161^Tb). Notably, ^177^Lu serves as a β‐ and γ‐emitter, whereas ^161^Tb is an Auger and β‐emitter; both are utilized in therapy and SPECT imaging. Peptides were synthesized via solid‐phase peptide synthesis. Cytotoxicity assays in T98 glioblastoma cells showed that Angiopep‐2 is well‐tolerated, maintaining ~100% viability at 20 μM and a moderate decline up to 100 μM. Radiolabeling achieved yields > 95% with excellent radiochemical stability at room temperature for up to 10 days and moderate stability in the presence of human serum. Biodistribution in healthy CFW mice showed a brain‐associated radioactivity of 0.24% ± 0.05% IA/g at 5 min p.i. and a 12‐fold increase in the brain‐to‐blood ratio (0.028–0.339) by 60 min p.i. These results support DOTA‐Angiopep‐2 as a versatile platform for radionuclide delivery and a potential candidate for future glioma‐targeted studies. Further studies in tumor‐bearing models are ongoing to evaluate therapeutic efficacy and translational potential.

## Graphical abstract

The developed DOTA‐Angiopep‐2 conjugate was well tolerated in glioma cells and enabled high‐yield radiolabeling with ^177^Lu and ^161^Tb. The ^161^Tb conjugate showed brain delivery with predominantly renal clearance, supporting a stable radiotheranostic platform for BBB‐targeted glioma therapy and SPECT imaging.

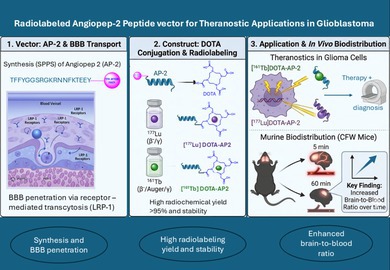

## 1 Introduction

The central nervous system (CNS) is protected by the blood–brain barrier (BBB), a highly selective and dynamic interface that restricts the entry of most therapeutic and diagnostic agents into the brain parenchyma. This presents a significant obstacle in the treatment and imaging of brain tumors. In addition to this challenge, the tumor microenvironment (TME) of gliomas introduces further barriers to drug delivery, including enhanced interstitial pressure, dense extracellular matrix components, immunosuppressive signaling, and a heterogeneous cellular landscape comprising cancer stem cells, immune cells, and stromal elements. These hinder the effective accumulation and uptake of therapeutic compounds within the tumor. To address these limitations, receptor‐mediated targeting approaches have emerged as a compelling strategy, offering the potential to bypass the BBB and selectively navigate the TME. Such methods represent a promising strategy for enhancing the delivery of compounds that may eventually improve therapeutic outcomes in CNS disorders [1]. Peptide‐based drug delivery systems have shown great promise in overcoming this challenge [1, 2, 3]. Peptides have emerged as versatile delivery vectors for therapeutic agents across various malignancies, including brain cancer, and are increasingly investigated for crossing the BBB [4]. They can traverse the BBB via multiple pathways, including receptor‐mediated transcytosis, adsorptive‐mediated transport, or through hybrid mechanisms that combine specific receptor targeting with efficient barrier penetration. This multifaceted transport potential makes peptides attractive candidates for CNS‐directed drug delivery strategies [1].

Angiopep‐2 (AP‐2) is a 19‐mer peptide (TFFYGGSRGKRNNFKTEEY) derived from aprotinin that crosses the BBB predominantly via receptor‐mediated transcytosis through interaction with the low‐density lipoprotein receptor‐related protein 1 (LRP1). This targeted mechanism enables efficient transport of AP‐2‐tethered agents into the CNS. LRP1 is overexpressed on both the BBB endothelial cells and glioma cells, positioning it as an attractive target for CNS drug delivery. Beyond its role in endocytosis, LRP1 contributes to cellular signaling and vascular homeostasis, reinforcing its relevance within the TME. By harnessing LRP1‐mediated transport, AP‐2 serves as a potential scaffold for the delivery of diagnostic and therapeutic payloads across the BBB, addressing one of the primary physical obstacles in glioma research [5].

Upon intravenous administration, AP‐2 tethered to nanoparticles has been shown to bind to LRP1 receptors expressed on both endothelial and glioma cells. This interaction facilitates receptor‐mediated endocytosis and transcytosis, which are the biological mechanisms intended to support the transport and internalization of payloads within the CNS [5, 6, 7, 8]. Also, AP‐2 has been conjugated with imaging agents and paclitaxel (ANG1005), with the latter being used in clinical trials [9]. Although AP‐2's capacity to transport diverse molecular cargoes into the brain has been well established, its potential to be exploited as a radiotheranostic platform capable of both imaging and therapy through radiometal conjugation that could unlock its full utility in precision oncology remains largely underexplored. Initial radiolabeling studies of AP‐2 utilized iodine‐125, which remains the most commonly used radionuclide due to its straightforward chemistry for peptide labeling in early studies [10]. However, the broader field of radiotheranostics is increasingly shifting toward the use of radiometals, which form the basis for modern precision oncology. Radiometals can be categorized as positron (β^+^) and gamma (γ) emitters and are commonly applied in imaging techniques such as positron emission tomography (PET) and single‐photon emission computed tomography (SPECT) [11]. On the other hand, therapeutic emitters (α‐particles, β^−^‐particles, or Auger electrons) exert cytotoxic effects through localized radiation [12, 13, 14]. In the context of AP‐2, the transition from radioiodine isotopes like ^125^I to radiolanthanides such as ^177^Lu and ^161^Tb represents a major leap in therapeutic efficiency. This is primarily due to superior metabolic stability and residualizing properties; although iodinated peptides are frequently deiodinated in vivo and leak from the cell after metabolism, DOTA‐conjugated radiometals remain trapped within the lysosomal compartment postinternalization, maximizing the intracellular residence time. Among these radiometals, Lutetium‐177 (^177^Lu) and Terbium‐161 (^161^Tb) are the most promising candidates for AP‐2‐based therapy. ^177^Lu is a beta‐particle emitter with a half‐life of 6.7 days and a maximum beta energy of 497 keV. This radionuclide is an ideal candidate for radiotheranostic purposes. It emits β^−^ particles, which are suitable for targeted therapy when conjugated to an appropriate targeting vector, and γ‐photons, which enable SPECT imaging [15, 16]. The use of ^177^Lu in the radiopharmaceuticals industry has greatly increased over the last years in therapeutic agents approved for human use, such as Lutathera and Pluvicto, which are used for the therapy of neuroendocrine tumors and prostate cancer, respectively [15]. Moreover, ^177^Lu decays to stable Hafnium‐177 (^177^Hf), enhancing radiation safety [17]. The combination of therapeutic and diagnostic applications makes ^177^Lu a key isotope in the radiotheranostics field.

On the other hand, ^161^Tb is a very promising radionuclide that is consistently gaining interest in the field of targeted radionuclide therapy. It is a beta‐emitter (*E* = 154 keV) with a half‐life of 6.96 days, which decays to Dysprosium‐161 (^161^Dy). Ιn addition, ^161^Tb emits photons, so it can be considered a very promising candidate for SPECT imaging. At the same time, it emits a considerable number of Auger electrons and conversion electrons with a high linear energy transfer over a short distance (< 40 keV/μm). This enables precise radiation deposition within tumor cells, improving the extent of DNA damage and therapeutic effectiveness. These electrons enable highly localized subcellular irradiation, unlike beta particles. The decay characteristics and properties are actually very similar to those of ^177^Lu (*t*_1/2_ = 6.65 days, *E*_β−_ = 134 keV; *E*_γ_ = 113 keV, *E*_γ_ = 208 keV), but the co‐emission of conversion and Auger electrons further improves its radiotheranostic potency. Moreover, the presence of low‐energy gamma emission in ^161^Tb, compared with ^177^Lu, may offer certain practical advantages in radiation protection [18]. ^161^Tb is recognized as a cutting‐edge radiotheranostic radiometal, combining precise cellular targeting, both imaging and therapeutic functions, and strong potential for rapid clinical translation [19, 20, 21]. Consequently, although ^125^I is still useful for pharmacokinetic tracking, the current terbium era offers a significantly higher therapeutic index for BBB‐penetrating vectors.

The efficacy of a radionuclide‐based agent largely depends on the stability of the radiometal–chelator complex, and along these lines, appropriate chelating agents are employed [15]. In this framework, chelators such as 1,4,7,10‐tetraazacyclododecane‐1,4,7,10‐tetraacetic acid (DOTA), 2,2′‐([2‐(bis(carboxymethyl)amino)ethyl]azanediyl)bis(methylene)diacetic acid (DTPA), or 1,4,7‐triazacyclononane‐1,4,7‐triacetic acid (NOTA) have been utilized to form stable complexes [22, 23, 24]. Among them, DOTA, a polyaminocarboxylic acid, is widely used because of its strong affinity for different radiometals and the radiostability of the resulting complexes. It has been used for conjugating diagnostic and therapeutic radionuclides like ^68^Ga, ^64^Cu, ^177^Lu, and ^161^Tb [18, 24].

As highlighted above, AP‐2 has not been extensively explored in the context of radiotheranostics. To the best of our knowledge, the potential of DOTA‐conjugated AP‐2 radiolabeled with therapeutic radionuclides such as ^177^Lu and ^161^Tb has yet to be investigated. Building upon this gap, the present study focuses on the design, radiochemical synthesis, and preliminary evaluation of an AP‐2‐based platform for radiometal conjugation. Toward this, two novel peptide‐based constructs were synthesized, consisting of the BBB‐penetrating carrier AP‐2, the radiometal chelator DOTA, and the radiotheranostic radionuclides ^177^Lu and ^161^Tb. The resulting conjugate, DOTA‐AP‐2, was evaluated for its radiochemical stability and its initial biodistribution profile in healthy mice. The combination of high radiolabeling efficiency, serum stability, and low cytotoxicity establishes DOTA‐AP‐2 as a chemically viable scaffold. Although further evaluation in brain tumor models is required, these findings provide a preliminary basis for the investigation of AP‐2‐based vectors in CNS‐targeted radiometal delivery.

## 2 Materials and Methods

All Fmoc‐protected amino acids, Rink amide resin, 1‐hydroxybenzotriazole (HOBt), and 1‐[bis(dimethylamino)methylene]‐1*H*‐1,2,3‐triazolo[4,5‐*b*]pyridinium 3‐oxid hexafluorophosphate (HATU) were purchased from GL Biochem (China). *N,N′*‐Diisopropylcarbodiimide (DIC), triisopropylsilane (TIS), *N,N*‐diisopropylethylamine (DIPEA), and piperidine were supplied by Merck‐Schuchardt (Germany). Dimethylformamide (DMF), trifluoroacetic acid (TFA), diethyl ether, Trizma base, 2‐(4,7,10‐tris(2‐(tert‐butoxy)‐2‐oxoethyl)‐1,4,7,10‐tetraazacyclododecan‐1‐yl) acetic acid (DOTA‐tris‐tBu ester), and iodoacetic acid were purchased from Sigma‐Aldrich. Acetonitrile was purchased from CARLO ERBA (Italy). DCM and hexane were supplied by Fisher Scientific (UK). All reagents and solvents used for the radiolabeling studies had a purity of > 95% and were obtained from commercial sources without further purification. Water was deionized to 18 MΩ·cm using an Easypure water filtration system (Barnstead International, Dubuque, IA, USA). Additionally, ultra‐trace free water, tri‐sodium citrate dehydrate (≥ 99.5%), and methanol (≥ 99.8%) were purchased from Fisher Scientific (Leicestershire, UK). Moreover, ethanol absolute and ammonium acetate were obtained from Carlo‐Erba (Val‐de‐Reuil, France). Human serum and sodium acetate (≥ 99.995%) were purchased from Sigma Aldrich (St. Louis, MO, USA). Protein LoBind Tubes (1.5 mL) were obtained from Eppendorf SE (Hamburg, Germany).

^161^Tb was provided as [^161^Tb]TbCl_3_ by TerThera (Breda, The Netherlands), whereas ^177^Lu was provided as [^177^Lu]LuCl_3_ by ITM Isotope Technologies Munich SE. Radioactivity was measured using a dose calibrator (Capintec, Ramsey, NJ, USA). Glass microfiber chromatography paper impregnated with silica gel (ITLC‐SG) was purchased from Agilent Technologies (Santa Clara, CA, USA) and was used in the determination of radiolabeling yield during radiolabeling and stability studies on a radio‐TLC scanner (Scan‐Ram, LabLogic, Sheffield, UK). Data collection and analysis were performed with Laura software v. 5.0.4.29.

For animal experiments, Carworth Farms White (CFW) mice of both genders were used. The mice were housed in individually ventilated cages (IVCs) with constant temperature (22°C ± 2°C) and humidity (45%–50%) and a 12‐h light/dark cycle, with free access to food and water. Our experimental animal facility is registered according to Greek Presidential Decree 56/2013 (Reg. Number: EL 25 BIO 022), in accordance with European Directive 2010/63, which is harmonized with national legislation on the protection of animals used for scientific purposes. All applicable national guidelines for the care and use of animals were followed. The study protocol was approved by the Department of Agriculture and Veterinary Service of the Prefecture of Athens. These studies have been further approved by our institutional ethics committee, and the procedures followed are in accordance with institutional guidelines. Samples for ex vivo biodistribution studies were measured on a Packard COBRA II Auto‐Gamma Counter (Ramsey, MN, USA).

### 2.1 Synthesis of AP‐2 and Its DOTA Conjugate

AP‐2 was synthesized on a Rink amide AM resin according to the standard solid‐phase peptide synthesis (SPPS) and Fmoc/tBu strategy, as previously described [25]. The choice of the C‐terminal amide form of the peptide was based on our previous work, in which AP‐2 was successfully employed as a delivery vector for a therapeutic cargo in glioma models, demonstrating significant biological efficacy [8]. This prior evidence supports the use of the amidated variant as a functionally validated scaffold for brain‐targeted delivery applications. Furthermore, it can enhance proteolytic stability and mimic the native protein sequence. Amidation of the C‐terminus protects the peptide from degradation by serum carboxypeptidases, which is essential for maintaining the stability of the radiolabeled construct during systemic circulation and in vivo biodistribution. Additionally, the –CONH_2_ group neutralizes the negative charge associated with a free carboxylate, providing a closer representation of the electronic state of the AP‐2 sequence within its parent molecule, aprotinin, which may influence its binding affinity and transcytosis efficiency via the LRP1 receptor. The full process includes sequential reactions of Fmoc‐group (9‐fluorenylmethyloxycarbonyl) removal and coupling of each amino acid with intervening washing with the solvents *N,N*‐DMF and dichloromethane (DCM). Deprotection of the Να‐group was achieved by applying a solution of 20% piperidine in DMF. The amino acid couplings were performed by adding the preactivated Fmoc‐Xaa to the resin and allowing the reaction to proceed for 3 h. The deprotection and coupling steps were monitored by the Kaiser test. DOTA was coupled with AP‐2 on the solid phase, following the previously reported procedure, slightly modified [26]. Specifically, DOTA‐tris(tBu) ester was dissolved in a 1:1 (v/v) mixture of DMF and DCM and preactivated with DIC and HOBt for 30 min. The activated solution was then added to the reaction vessel containing the preswelled peptide‐resin (in DMF/DCM) and allowed to react for 3 h. This coupling step was repeated three times to maximize the conjugation efficiency of DOTA‐tris(tBu) ester. Following the reaction, the resin was thoroughly washed with DMF and DCM and subsequently dried under vacuum. The peptide‐resin was treated with a cleavage cocktail consisting of TFA, TIS, and water in a ratio of 95:2.5:2.5 (v/v/v). The cleavage reaction was allowed to proceed for 4 h at room temperature (RT). After completion, the filtrate was collected, and the solvents were evaporated under reduced pressure. The crude peptide was precipitated by the addition of cold diethyl ether, followed by filtration. The resulting solid was dissolved in slightly acidified water, and the solution was subsequently lyophilized to yield the crude product.

**FIGURE 1:**
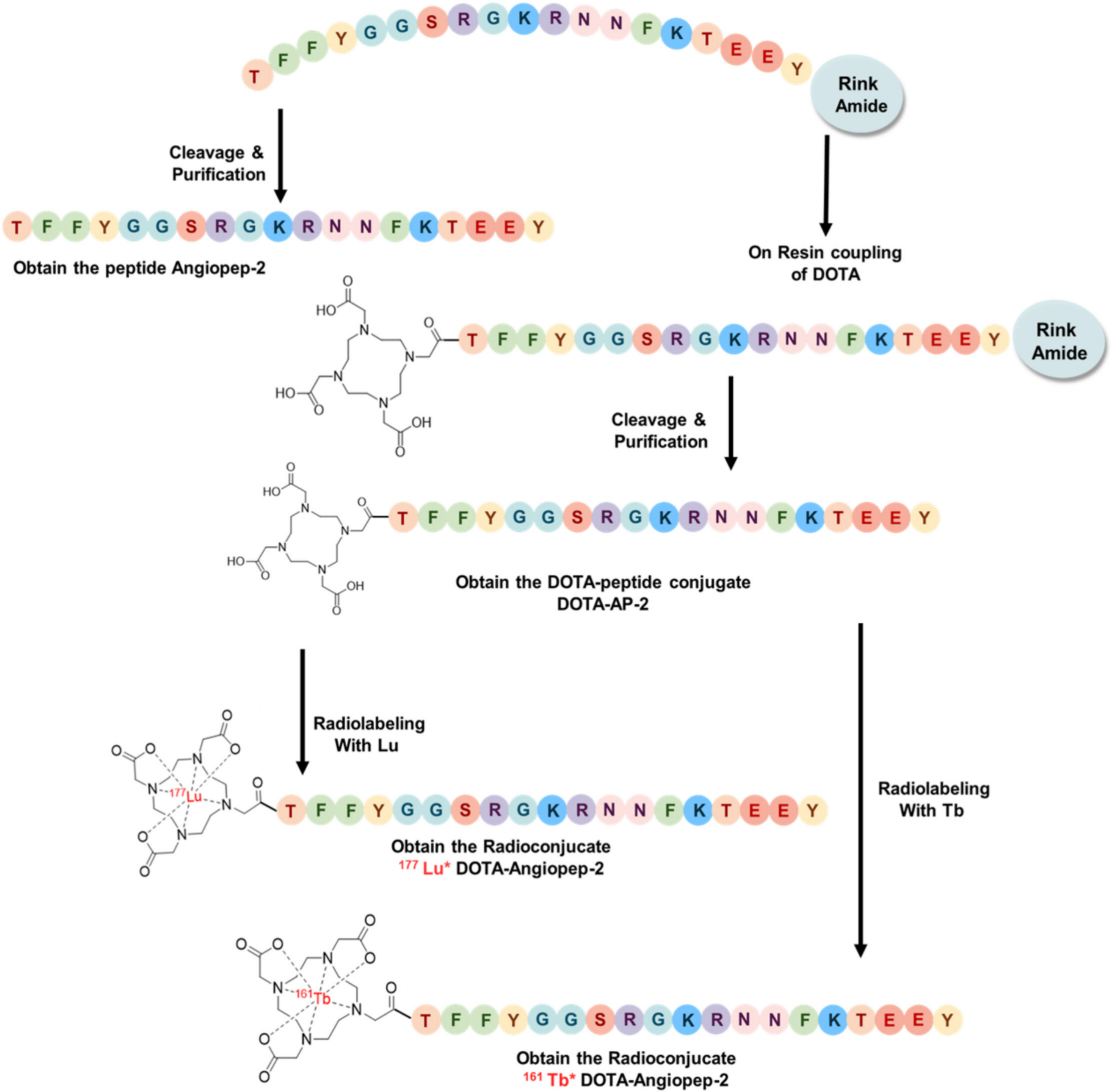
Synthetic steps for the synthesis of AP‐2, DOTA‐AP‐2 conjugate, and the radiolabeling of DOTA‐AP‐2 with ^177^Lu and ^161^Tb.

The synthetic procedure is illustrated in Figure 1.

### 2.2 Characterization of AP‐2 and the DOTA‐AP‐2 Conjugate

AP‐2 and DOTA‐AP‐2 conjugates were purified by RP‐HPLC with gradient elution methods. The mobile phases consisted of Solvent A: water containing 0.1% TFA, and Solvent B: acetonitrile containing 0.1% TFA. The chromatographic separation was monitored at 214 nm over a 30‐min injection run. The gradient was from 90% A/10% B to 10% A/90% B, allowing effective resolution and isolation of the target peptides. The column was Jupiter Phenomenex (4 μm Proteo 90 Å, 250 × 10 mm). The MW of the synthesized compounds was confirmed via mass spectrometry using a quadrupole time‐of‐flight (QToF) system, operated in positive electrospray ionization (ESI) mode. Prior to analysis, the samples were reconstituted in LC–MS grade water. To evaluate further the purity of each compound, analytical RP‐HPLC was performed under the same solvent gradient as used during purification, over 20 min. The Poroshell 120 (EC‐C18 4 μm, 4.4 × 100 mm) analytical column was utilized.

### 2.3 Evaluation of Cytotoxicity of AP‐2

T98 glioblastoma cells were cultured in DMEM supplemented with 10% FBS and antibiotics. Approximately 20,000 cells/well were seeded in 12‐well plates and allowed to adhere for 24 h. Cells were then treated with AP‐2 at concentrations of 20, 40, 60, 80, and 100 μM and incubated for 72 h. Cell viability was assessed using the trypan blue exclusion assay, and results were evaluated by calculating the mean viable cell percentage in comparison to the untreated control. The percentage of viability and the concentration that causes 50% of cell death, or IC_50_, were calculated. We utilized nonlinear regression analysis with GraphPad Prism software (v. 8.0.0, San Diego, CA, USA, Trial Version) to calculate the IC50 value. All experiments were performed in triplicate.

### 2.4 Radiolabeling Studies of DOTA‐AP‐2

Radiolabeling studies of DOTA‐AP‐2 with ^177^Lu and ^161^Tb were necessary to evaluate the yield of radiometal conjugation to the theranostic platform. The radiolabeling procedure is straightforward. Briefly, DOTA‐AP‐2 was dissolved in trace‐free H_2_O to a final concentration of 1 mg/mL. Then, 54 μL (20 nmol) was added to 300 μL of trace‐free sodium acetate buffer (pH 4.9). Subsequently, 5–10 μL of [^161^Tb]TbCl_3_ or [^177^Lu]LuCl_3_ (~10–30 MBq/radiolabeling) was also added, and the final reaction mixture was incubated at 90°C for 1 h.

The radiolabeling yield was evaluated by instant thin layer chromatography (ITLC) using silica gel sheets and sodium citrate 0.1 M as mobile phase. Also, for the determination of colloids, ammonium acetate:MeOH (1:1) was used. Ten microliters of radiolabeled DOTA‐AP‐2 was applied to the application point of the ITLC‐SG strip and was left to dry. In the sodium citrate system, the free ^177^Lu^3+^ or ^161^Tb^3+^ migrates to the front (*R*_*f*_ = 0.8–1.0), leaving the radiolabeled compound at the origin (*R*_*f*_ = 0.0–0.2). Additionally, in the ammonium acetate:MeOH system, the radiolabeled compound migrates to the front (*R*_*f*_ = 0.8–1), leaving the colloids strictly at the origin (*R*_*f*_ = 0.0–0.2).

### 2.5 In Vitro Stability Studies of [^177^Lu]Lu‐DOTA‐AP‐2 and [^161^Tb]Tb‐DOTA‐AP‐2

The in vitro stability studies were conducted to evaluate the radiochemical robustness of [^177^Lu]Lu‐DOTA‐AP‐2 and [^161^Tb]Tb‐DOTA‐AP‐2. These experiments determine whether the peptide and the radiolabel remain intact under biologically relevant conditions, identify potential degradation, and provide data needed to interpret biodistribution results and to support selection of formulation, dosing, and further preclinical development. In vitro stability was assessed at RT (bench stability). Since [^161^Tb]Tb‐DOTA‐AP‐2 was to be administered to mice, its stability in serum was examined prior to administration. In particular, radiolabeled DOTA‐AP‐2 was added to human serum at a ratio of 1:1 and was incubated at 37°C for up to 10 days postradiolabeling. The evaluation was performed with ITLC‐SG using sodium citrate 0.1 M and ammonium acetate (1 M): MeOH as mobile phases. All experiments were performed in triplicate.

### 2.6 Biodistribution Studies of [^161^Tb]Tb‐DOTA‐AP‐2

In vivo biodistribution studies were performed in murine models to elucidate the pharmacokinetics and tissue‐specific accumulation of [^161^Tb]Tb‐DOTA‐AP‐2 for evaluating its potential for the preliminary characterization of its pharmacokinetic profile and systemic distribution. In vivo kinetics of [^161^Tb]Tb‐DOTA‐AP‐2 were evaluated in 6‐ to 8‐week‐old Carworth Farms Webster (CFW) mice, weighing 25–30 g. The animals were kept in IVCs with a 12‐h light/dark cycle and had unrestricted access to food and water. The radiolabeled sample was reconstituted in ~1000‐μL final volume with saline and 10% absolute ethanol. A 100‐μL dose of 550–600 kBq was intravenously administered to each mouse. At 5 and 60 min postadministration, the mice were euthanized by isoflurane inhalation, and the major organs and tissues of interest were collected, weighed, and measured in an automatic gamma counter. The animals were not saline‐perfused before brain collection. The syringes containing the radiolabeled peptide were measured full and empty to accurately determine the administered dose for each mouse. In order to calculate the injected dose per animal, a standard solution was prepared, and the tail activity was subtracted. All measurements were adjusted for background radiation. The biodistribution data were expressed as percentage of injected activity per gram of tissue (%IA/g).

## 3 Results and Discussion

### 3.1 Synthesis and Characterization of AP‐2 and DOTA‐AP‐2

**FIGURE 2:**
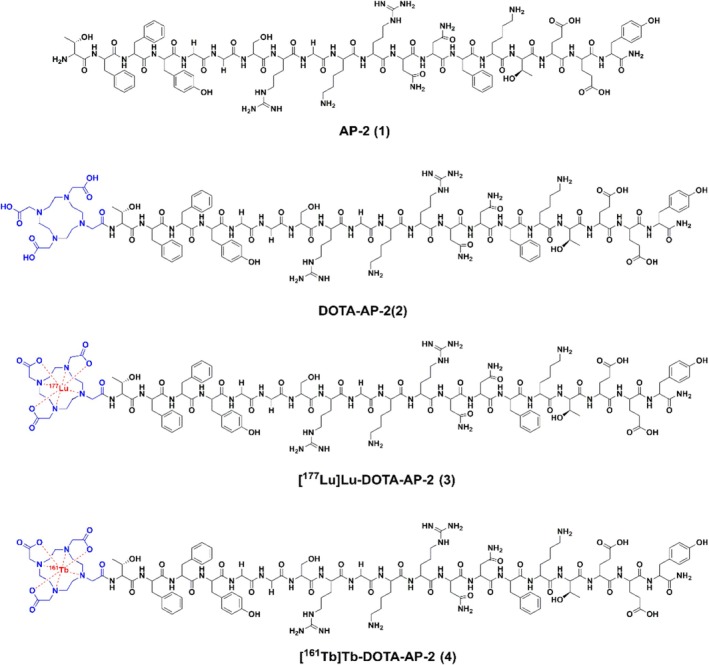
Structures of the four synthesized compounds: (**1**) AP‐2, (**2**) DOTA‐AP‐2, (**3**) [^177^Lu]Lu‐DOTA‐AP‐2, and (**4**) [^161^Tb]Tb‐DOTA‐AP‐2.

AP‐2 and its derivative, DOTA‐AP‐2 (Figure 2), were successfully synthesized by SPPS. The identity and purity of both compounds were confirmed via ESI‐MS (QToF) and RP‐HPLC, respectively. Figures S1 and S3 reveal the presence of the [M+2H^+^]/2, [M+3H^+^]/3, [M+4H^+^]/4, and [M+5H^+^]/5 ion species of AP‐2 and DOTA‐AP‐2, respectively, whereas Figures S2 and S4 illustrate the chromatograms of analytical HPLC of these two compounds. Taken together, the combination of mass spectrometric data and analytical chromatography confirms the successful synthesis and high purity of AP‐2 and its DOTA‐conjugated derivative (Figure 2), providing a solid basis for further radiolabeling and biological evaluation.

### 3.2 Cytotoxicity Evaluation of AP‐2

**FIGURE 3:**
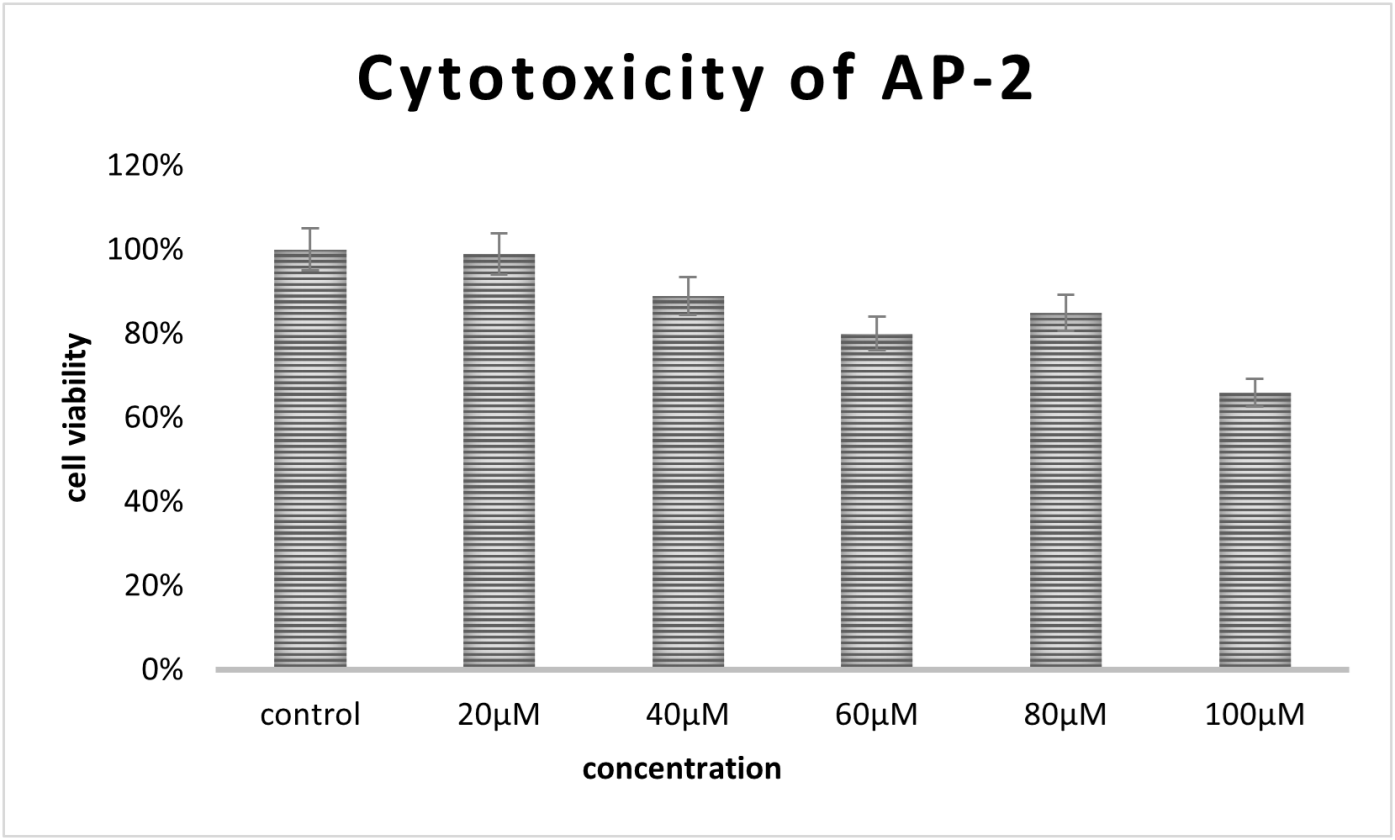
Graphical representation of cytotoxic effect in T98 cells after AP‐2 treatment.

Following the characterization and purity assessment of AP‐2, we evaluated its potential cytotoxicity in glioma cells. Along these lines, we selected the T98 glioblastoma cell line to evaluate it since it is widely recognized for its high resistance to the classically used glioma chemotherapeutic temozolomide. This resistance is mainly attributed to enhanced levels of the DNA repair enzyme MGMT, which restores the temozolomide‐induced DNA damage. T98 cell viability following treatment with AP‐2 remained high at 20 μM (~100%) and decreased moderately at higher concentrations (60–100 μM), as illustrated in Figure 3. The IC_50_ value was determined to be 58.5 μΜ. These results indicate that AP‐2 is generally well tolerated at lower concentrations but exhibits a mild dose‐dependent viability decline. Cytotoxicity data confirm the safety profile of the AP‐2 carrier, supporting its further evaluation as a brain targeting vector in therapeutic or diagnostic formulations.

### 3.3 Radiolabeling Studies of DOTA‐AP‐2 With ^177^Lu and ^161^Tb

**FIGURE 4:**
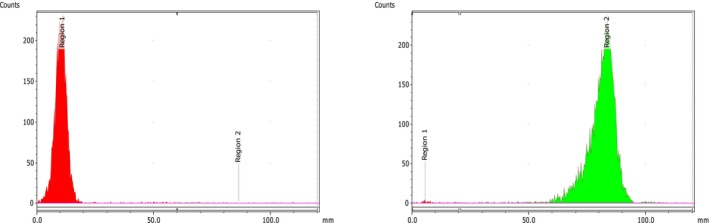
Radiochemical purity data of [^161^Tb]Tb‐DOTA‐AP‐2 using sodium citrate of 0.1 M as a mobile phase (left panel: no degradation observed: *R*_*f*_ of radiolabeled compound, 0.0–0.2) and ammonium acetate:methanol (1:1) (right panel: insignificant amount of radiocolloids detected: *R*_*f*_ of radiocolloids, 0.0–0.2).

The radiolabeling procedure previously described achieved excellent yields, exceeding 95% after only 1 h incubation at 90°C. The evaluation was carried out using ITLC‐SG and a radio‐TLC scanner or gamma‐counter. The results are presented below (in Section 3.4). This high radiolabeling profile is attributed to the strong chelating properties of DOTA, which forms a very strong complex with both radiometals under mild conditions. The negligible free radiometals indicate a high radiolabeling yield (Figure 4). The radiolabeled peptide and free ^161^Tb chloride migrate to a characteristic *R*_*f*_ position, allowing them to be distinguished spatially on the chromatogram. RCP was calculated by integrating the counts (peak areas) corresponding to the radiolabeled product peak and the total radioactivity on the strip (sum of all peaks, including free metal). The RCP is then calculated as

$$\mathrm{RCP}\;\left(\%\right)=\frac{\text{Activity of radiolabeled product}}{\text{Total activity}\;\mathrm{on}\;\text{strip}}\times 100\%$$

For instance, the radio‐iTLC analysis demonstrated that free ^161^Tb accounted for 0.90% of the total activity, whereas radiocolloids contributed 1.11%. Consequently, the fraction corresponding to the radiolabeled peptide was calculated by difference, yielding a radiochemical purity (RCP) of 97.99% (100.00% − 0.90% − 1.11% = 97.99%). Following the successful radiolabeling, stability studies were also carried out to assess the robustness of both radiolabeled peptides under several conditions.

### 3.4 In Vitro Stability Studies of [^177^Lu]Lu‐DOTA‐AP‐2 and [^161^Tb]Tb‐DOTA‐AP‐2

**FIGURE 5:**
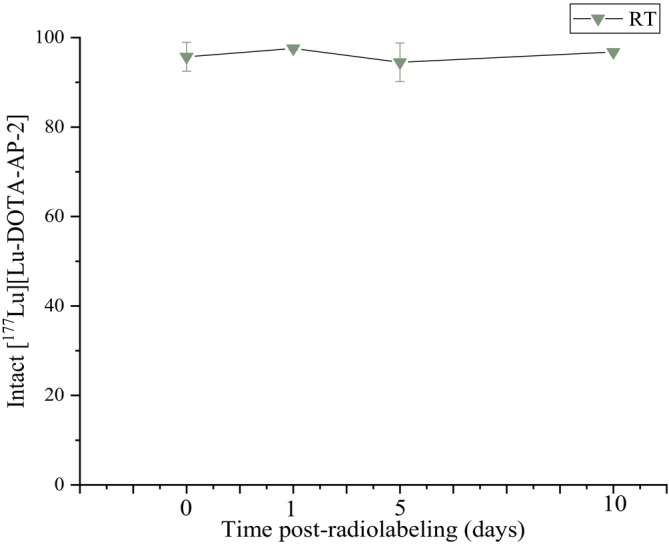
Radiochemical stability data of [^177^Lu]Lu‐DOTA‐AP‐2 at room temperature (bench stability) for up to 10 days (*x*‐axis not in scale). Day 0 represents the radiolabeling purity of the compound immediately after the radiolabeling procedure.

**FIGURE 6:**
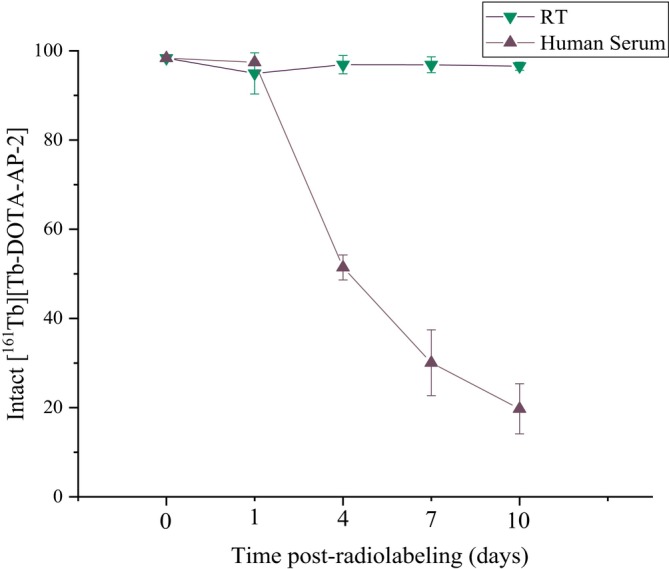
Radiochemical stability data of [^161^Tb]Tb‐DOTA‐AP‐2 at room temperature (bench stability) and in the presence of human serum for up to 10 days (*x*‐axis not in scale). Day 0 represents the radiolabeling purity of the compound immediately after the radiolabeling procedure.

In vitro stability studies for the two radioconjugates were performed at RT (Bench Stability), whereas the stability of [^161^Tb]Tb‐DOTA‐AP‐2 was also assessed in the presence of human serum at 37°C to determine the stability of the radiotracer in the presence of serum before its in vivo administration. The radiolabeled peptide showed satisfactory bench stability in both cases of radiometals (^177^Lu or ^161^Tb) at all timepoints examined (up to 10 days) (Figures 5 and 6).

**FIGURE 7:**
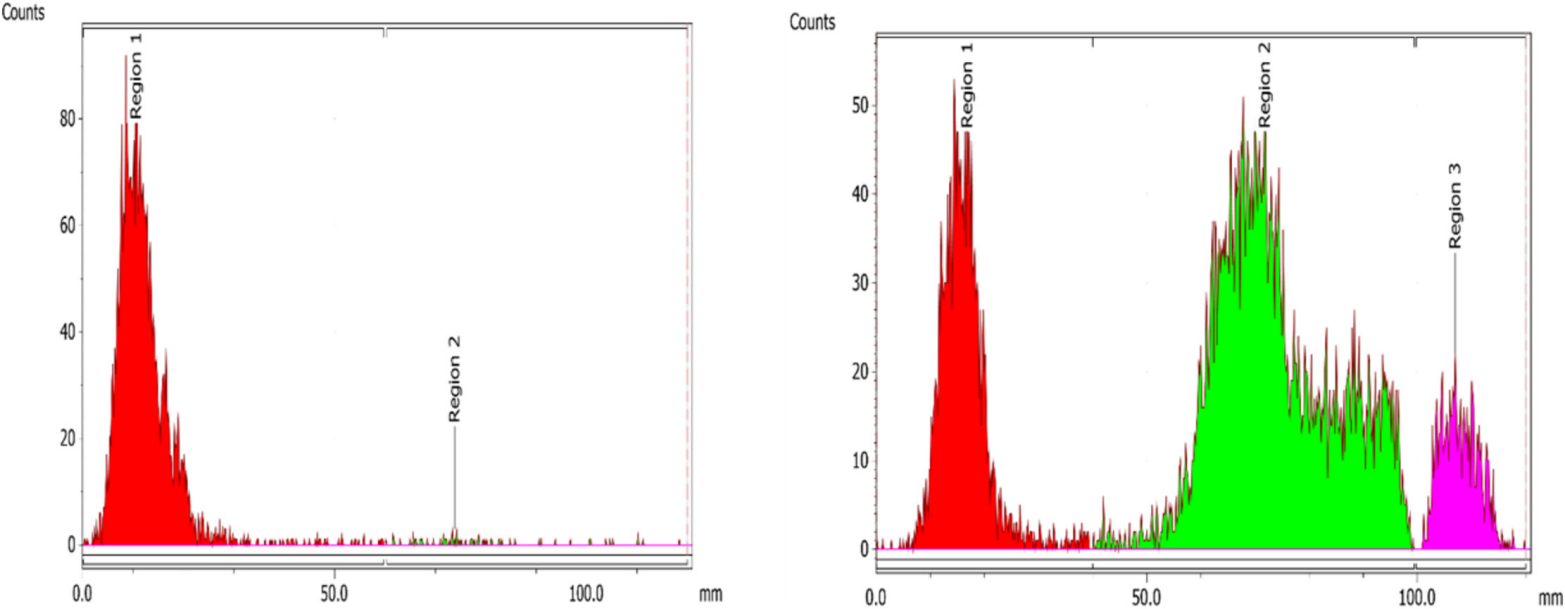
Radiochemical stability data of [^161^Tb]Tb‐DOTA‐AP‐2 in the presence of human serum using sodium citrate of 0.1 M as a mobile phase on Day 1 postradiolabeling (left panel: no degradation observed: *R*_*f*_ of radiolabeled compound, 0.0–0.2) and on Day 7 postradiolabeling (right panel: new peak observed: *R*_*f*_ = 0.5). For Day 10, the evaluation was carried out using a gamma‐counter due to the lower activity of the samples (peak counts attributed to degradation products: 78.40% ± 5.85%; intact radiolabeled peptide [^161^Tb]Tb‐DOTA‐AP‐2: 19.73% ± 5.62%).

In contrast, stability in human serum gradually decreased over time. The ITLC‐SG analysis indicated not only the presence of free ^161^Tb or colloids but also a new peak with *R*_*f*_ ~ 0.5, which gradually increased after Day 1 until Day 10 (Figure 7). This new peak may be attributed to a secondary radiochemical species consistent with degradation. This is commonly observed in the case of small peptides and is mainly due to the fact that the peptide is probably degraded by active proteases and peptidases present in serum. These enzymes recognize specific parts of the peptide and cleave the bonds between amino acids. As a result, the peptide gradually degrades. On the other hand, this phenomenon may also reduce the risk of prolonged accumulations in various tissues/organs and potential side effects [27, 28]. Radiolabeled species were identified only via radio‐iTLC, as radio‐HPLC analysis was not feasible. Thus, a definitive structural identification of the serum‐degraded product could not be provided.

### 3.5 Ex Vivo Biodistribution Studies of [^161^Tb]Tb‐DOTA‐AP‐2

**FIGURE 8:**
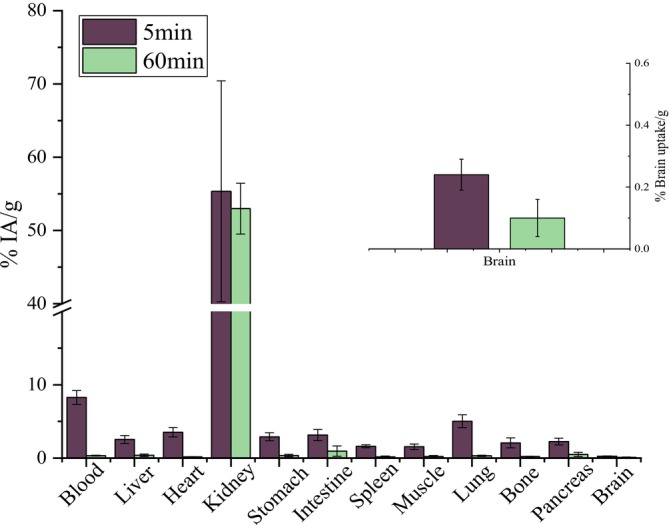
Ex vivo biodistribution results of [^161^Tb]Tb‐DOTA‐AP‐2 in CFW mice at 5 and 60 min postadministration. Mean values (*n* = 3) and SD (bars) are shown.

**FIGURE 9:**
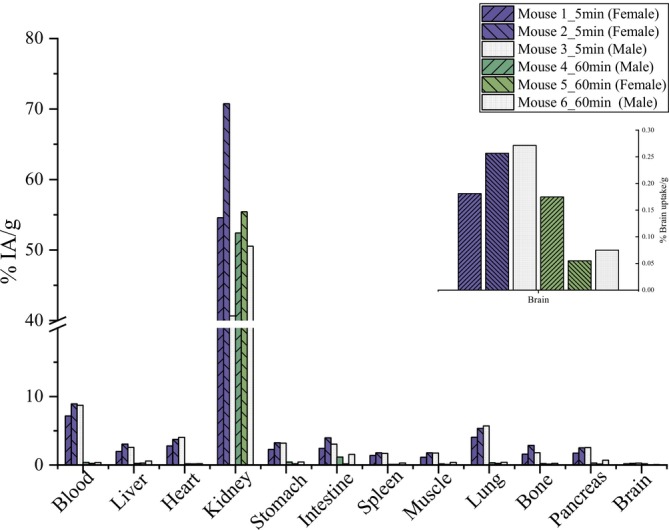
Ex vivo biodistribution results of [^161^Tb]Tb‐DOTA‐AP‐2 in mixed‐gender CFW mice at 5 and 60 min postadministration.

The radiolabeled peptide [^161^Tb]Tb‐DOTA‐AP‐2 was intravenously administered in normal mixed‐gender CFW mice for the determination of its in vivo behavior at 5 and 60 min postinjection. The results are presented below. As shown in Figure 8 and Table 1, the radiolabeled peptide presented very rapid blood clearance (from 8.27% ± 0.96% at 5 min p.i. to 0.33% ± 0.06% at 60 min p.i.) and high uptake in the kidneys, which remained stable at both examined timepoints. This is in accordance with other studies indicating that small peptides are mainly excreted via the renal pathway [29, 30]. Regarding our main topic of interest, notable brain‐associated radioactivity was observed at 5 min p.i. (0.24% ± 0.05%), which slowly decreased by 60 min p.i. (0.10% ± 0.06%). This finding provides a baseline for the peptide's behavior in vivo, considering the challenge of the BBB, which typically limits access to most therapeutic agents. Despite this gradual reduction, it is very important to point out that a twelvefold increase of the brain‐to‐blood ratio was observed over time, from 0.028 at 5 min to 0.339 at 60 min. This indicates that the clearance of the radiolabeled compound from the circulation is faster than from the brain, thus improving imaging contrast. All the other major organs presented very low uptake. The biodistribution results for each mouse are presented below (Figure 9).

**TABLE 1:** Biodistribution data for [^161^Tb]Tb‐DOTA‐AP‐2 at 5 and 60 min postadministration. Results are shown as the mean ± standard deviation (mean ± SD, *n* = 3).

Organ	5 min	60 min
Blood	8.27 ± 0.96	0.33 ± 0.06
Liver	2.54 ± 0.54	0.38 ± 0.18
Heart	3.53 ± 0.65	0.17 ± 0.02
Kidney	55.34 ± 15.07	52.99 ± 3.46
Stomach	2.91 ± 0.55	0.35 ± 0.16
Intestine	3.15 ± 0.77	0.95 ± 0.71
Spleen	1.61 ± 0.20	0.17 ± 0.11
Muscle	1.55 ± 0.37	0.21 ± 0.15
Lung	5.03 ± 0.87	0.31 ± 0.08
Bone	2.08 ± 0.68	0.20 ± 0.05
Pancreas	2.25 ± 0.46	0.50 ± 0.28
Brain	0.24 ± 0.05	0.10 ± 0.06

## 4 Conclusion

In this study, we present a rationally designed peptide‐based radiotheranostic platform, integrating three key components: the BBB‐penetrating peptide AP‐2, the DOTA chelator, and the radionuclides ^177^Lu and ^161^Tb. These radiometals were selected for their dual therapeutic and imaging capabilities, enabling the development of a modular and targeted system with potential utility in glioma research. Our results demonstrate the high radiolabeling stability of the DOTA‐AP‐2 platform, alongside preliminary brain‐associated radioactivity in nonperfused, healthy murine models. AP‐2 alone was well tolerated at lower concentrations, although DOTA‐AP‐2 and the corresponding metal complexes remain to be evaluated. The successful synthesis of AP‐2 and DOTA‐AP‐2, along with the radiolabeling of DOTA‐AP‐2 with ^177^Lu and ^161^Tb, and preliminary biological evaluation of ^161^Tb‐DOTA‐AP‐2 offer encouraging baseline data. These findings provide a preliminary radiochemical and biodistribution basis for further evaluation of AP‐2‐based radiometal conjugates in CNS‐targeted studies. Future studies aim to evaluate in‐depth the pharmacokinetics, tumor‐targeting efficiency, and therapeutic efficacy in relevant in vivo glioma models, with the long‐term goal of clinical translation in the field of neuro‐oncology.

## Funding

This project was financed by the National Recovery and Resilience Plan Greece 2.0, funded by the European Union–NextGenerationEU, under the call RESEARCH‐CREATE‐INNOVATE (project code: TAEDK‐06189/T2EDK‐0326, Acronym: Glioblastoma), https://greece20.gov.gr. Mr. Stavros Xanthopoulos is gratefully acknowledged for excellent technical assistance.

## Conflicts of Interest

The authors declare no conflicts of interest.

## Supporting information


**Figure S1:** Mass spectrum of the pure AP‐2. The ions with m/z 1151.1108, 767.7440, 576.0548, and 461.0479 correspond to the [M + 2H^+^]/2, [M + 3H^+^]/3, [M + 4H^+^]/4, and [M + 5H^+^]/5. Calculated MW: 2300.52 Da and measured MW: 2300.15 Da.
**Figure S2:** Chromatogram of analytical HPLC of the purified AP‐2. The retention time of the pure AP‐2 was observed at 5.55 min.
**Figure S3:** Mass spectrum of the pure DOTA‐AP‐2. The ions with m/z 1344.2477, 896.5005, 672.6273, and 538.3033 correspond to the [M + 2H+]/2, [M + 3H+]/3, [M + 4H+]/4, and [M + 5H+]/5. Calculated MW: 2686.93 Da and measured MW: 2686.52 Da.
**Figure S4:** Chromatogram of the analytical HPLC of the purified DOTA‐AP‐2. The retention time of the pure DOTA‐AP‐2 was measured at 5.39 min.

## Data Availability

The data that support the findings of this study are available on request from the corresponding authors.
