# Supplementary material for: Radiolabeled Angiopep‐2 Peptide Vector as a Preclinical Platform for Blood–Brain Barrier Targeting: Synthesis, Radiolabeling, and Preliminary In Vivo Biodistribution in Mice
Source: J Pept Sci. 2026 Jun 10;32(7):e70109. doi: 10.1002/psc.70109 (PMC13253176; doi:10.1002/psc.70109)
Supplement: Supplementary file 1 — Figure S1: Mass spectrum of the pure AP‐2. The ions with m/z 1151.1108, 767.7440, 576.0548, and 461.0479 correspond to the [M + 2H+]/2, [M + 3H+]/3, [M + 4H+]/4, and [M + 5H+]/5. Calculated MW: 2300.52 Da and measured MW: 2300.15 Da. Figure S2: Chromatogram of analytical HPLC of the purified AP‐2. The retention time of the pure AP‐2 was observed at 5.55 min. Figure S3: Mass spectrum of the pure DOTA‐AP‐2. The ions with m/z 1344.2477, 896.5005, 672.6273, and 538.3033 correspond to the [M + 2H+]/2, [M + 3H+]/3, [M + 4H+]/4, and [M + 5H+]/5. Calculated MW: 2686.93 Da and measured MW: 2686.52 Da. Figure S4: Chromatogram of the analytical HPLC of the purified DOTA‐AP‐2. The retention time of the pure DOTA‐AP‐2 was measured at 5.39 min. [file PSC-32-e70109-s001.docx]

**Supporting Information**

**Radiolabeled Angiopep-2 Peptide Vector as a Preclinical Platform for Blood-Brain Barrier Targeting: Synthesis, Radiolabeling and Preliminary In Vivo Biodistribution in Mice.**

Evgenia Fotou^1^, Adamantia Apostolopoulou^2^, Christina–Georgia Bika^1^, Maria Giannakopoulou^3^, Danai-Efraimia Bajwa^2^, Vassilios Moussis^1^, Vassilios Tsikaris^1^, Ioannis P Gerothanassis^1^, George A. Alexiou^3^, Penelope Bouziotis^2,*^, Andreas Tzakos^1,*^

^1^ Section of Organic Chemistry and Biochemistry, Department of Chemistry, University of Ioannina, 45110 Ioannina, Greece, [atzakos@uoi.gr](mailto:atzakos@uoi.gr)

^2^ Institute of Nuclear & Radiological Sciences & Technology, Energy & Safety, National Center for Scientific Research “Demokritos”, 15341 Athens, Greece, [bouzioti@rrp.demokritos.gr](mailto:bouzioti@rrp.demokritos.gr)

^3^ Neurosurgical Institute, University of Ioannina, 45110 Ioannina, Greece

Figure S1 displays the ESI-MS spectrum of AP-2, revealing a series of multiply charged ions at m/z values equal to 1151.1108, 767.7440, 576.0548, and 461.0479, which correspond to the [M+2H^+^]/2, [M+3H^+^]/3, [M+4H^+^]/4, and [M+5H^+^]/5 charge states, respectively. These signals correspond to a calculated MW of 2300.15 Da, which closely matches the theoretical MW of 2300.52 Da, indicating accurate synthesis and sequence identity of the peptide. To evaluate the purity of the peptide, analytical RP-HPLC was employed. As shown in figure S2, a well-shaped peak is depicted without any significant secondary peaks, which attests to the high purity of the synthesized compound. The retention time for the pure AP-2 was recorded at 5.55 min.

Similarly, the ESI-MS spectrum of DOTA-AP-2 (Figure S3) shows the ions with m/z 1344.2477, 896.5005, 672.6273, and 538.3033, which are consistent with the [M+2H^+^]/2, [M+3H^+^]/3, [M+4H^+^]/4, and [M+5H^+^]/5 species, respectively. The experimental MW value of 2686.52 Da aligns well with the theoretically calculated MW of 2686.93 Da, further confirming the successful incorporation of the DOTA moiety into the N-terminus of the AP-2 scaffold. Also, an additional peak with m/z 909.1502 Da was observed, which is ascribed to the [M+2H+K]^3+^/3 adduct. Potassium Adduct is commonly seen in ESI-MS, likely originating from trace ions in the solvents. Its appearance does not interfere with the primary molecular ion signals and can be considered a normal feature in mass analysis under positive ion mode conditions. The presence of multiple charge states in the mass spectra and the clear patterns validate the structural integrity, as well as the expected ionizing behaviour of the compounds. The purity of the conjugate was confirmed via RP-HPLC (Figure S4), where the retention time for DOTA-AP-2 was determined at 5.39 min. The earlier elution of the conjugate reflects the enhanced polarity imparted by the DOTA moiety.


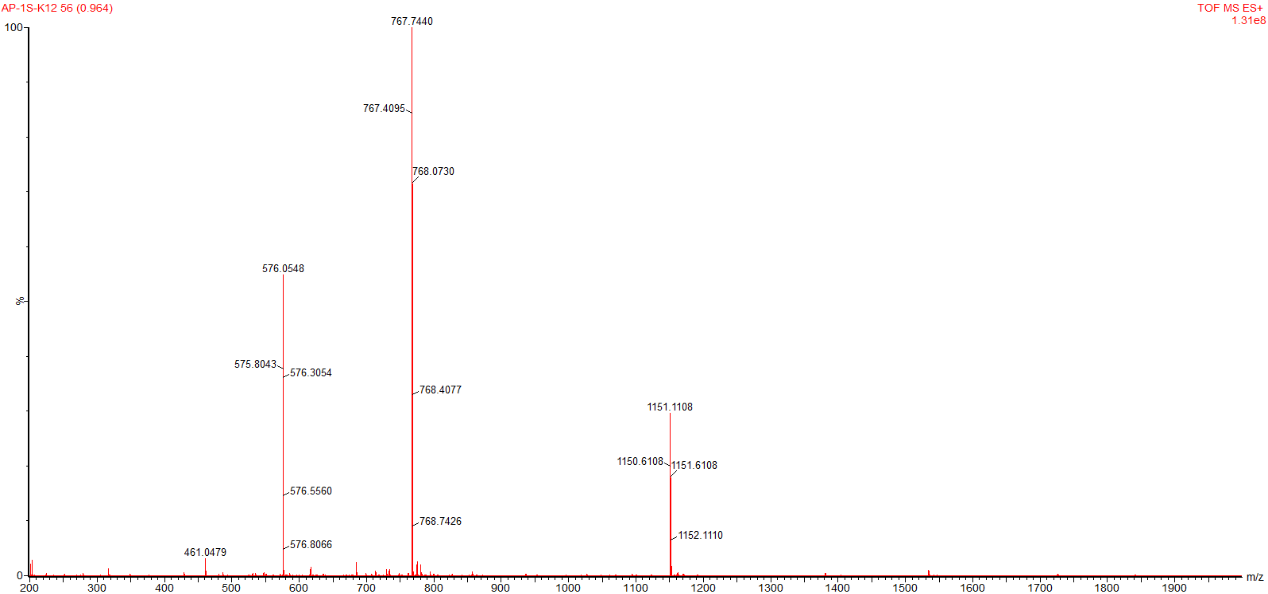


Figure S1. Mass spectrum of the pure AP-2. The ions with m/z 1151.1108, 767.7440, 576.0548, and 461.0479 correspond to the [M+2H^+^]/2, [M+3H^+^]/3, [M+4H^+^]/4, and [M+5H^+^]/5. Calculated MW: 2300.52 Da and measured MW: 2300.15 Da.


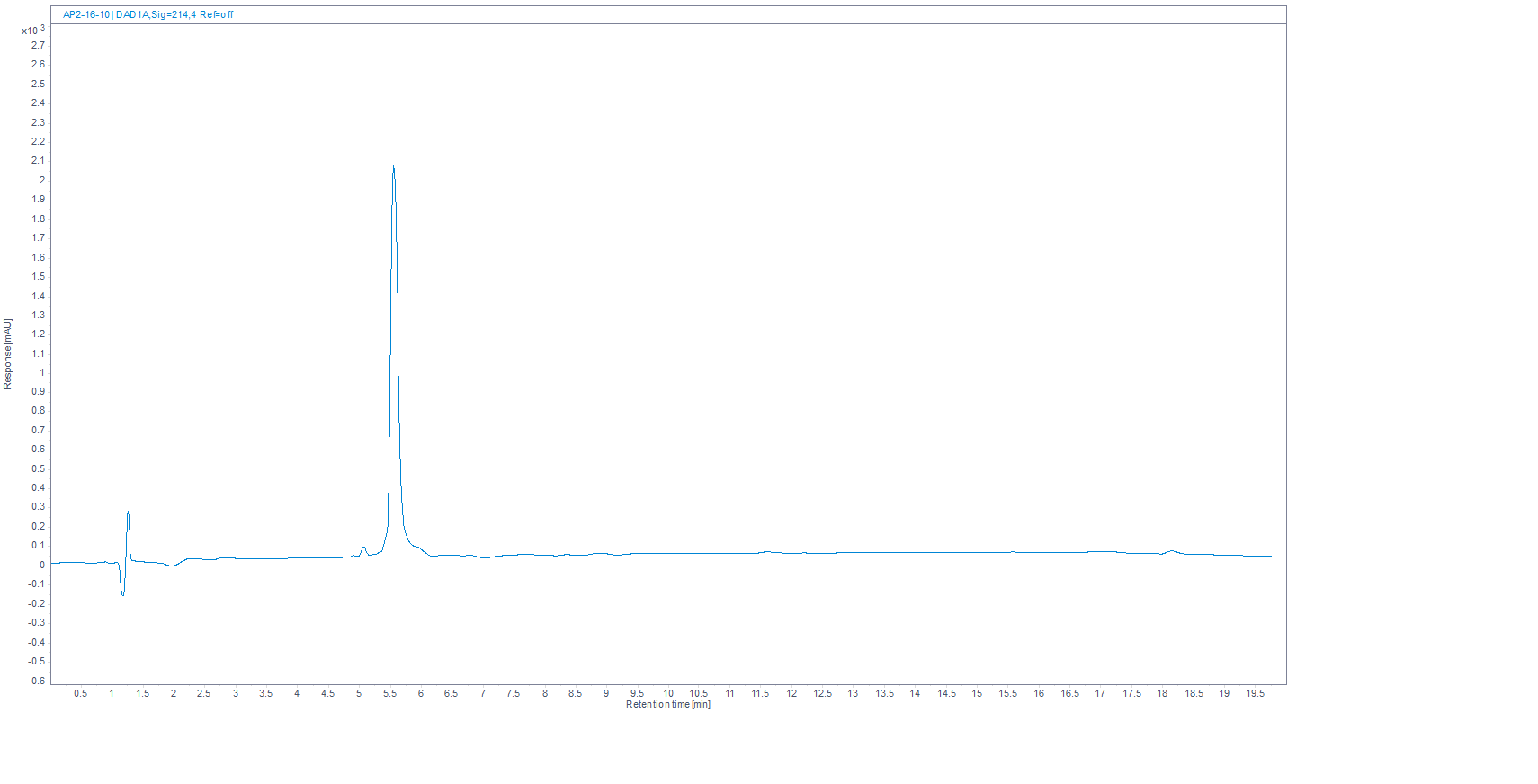
Figure S2. Chromatogram of analytical HPLC of the purified AP-2. The Retention Time of the pure AP-2 was observed at 5.55 min.


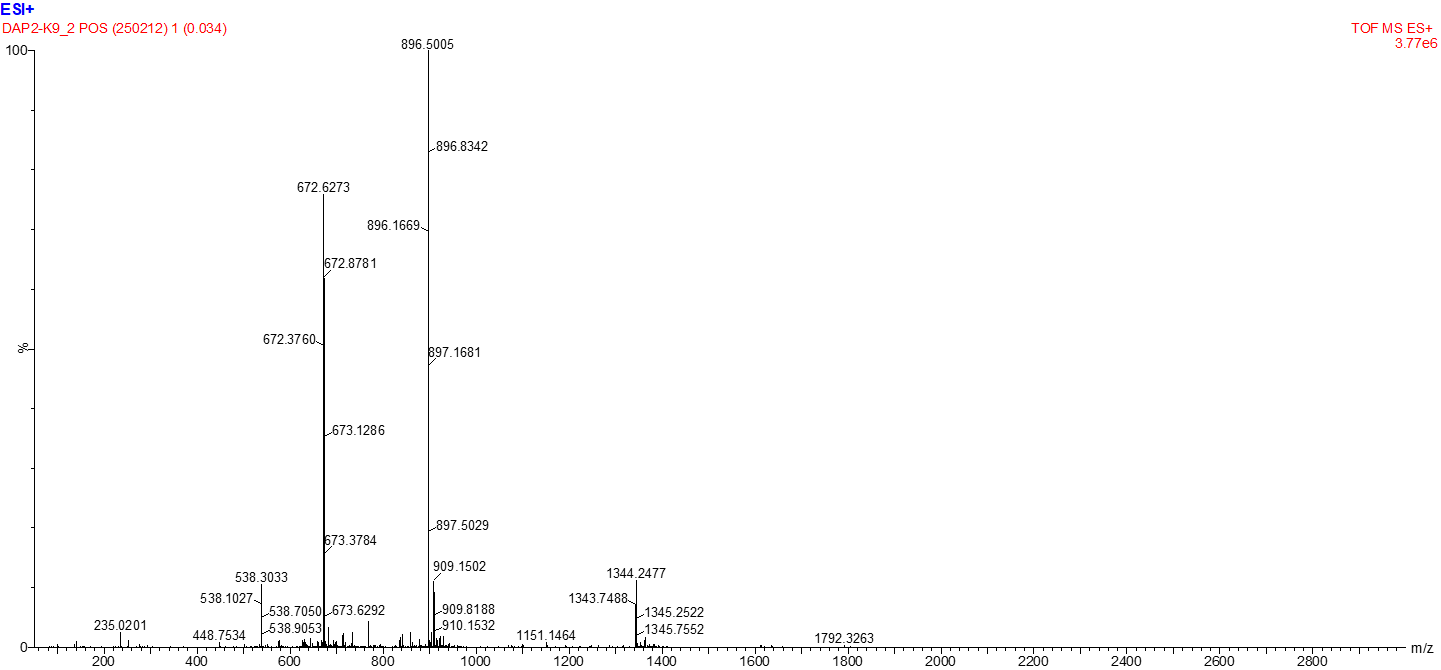


Figure S3. Mass spectrum of the pure DOTA-AP-2. The ions with m/z 1344.2477, 896.5005, 672.6273, and 538.3033 correspond to the [M+2H^+^]/2, [M+3H^+^]/3, [M+4H^+^]/4, and [M+5H^+^]/5. Calculated MW: 2686.93 Da and measured MW: 2686.52 Da.


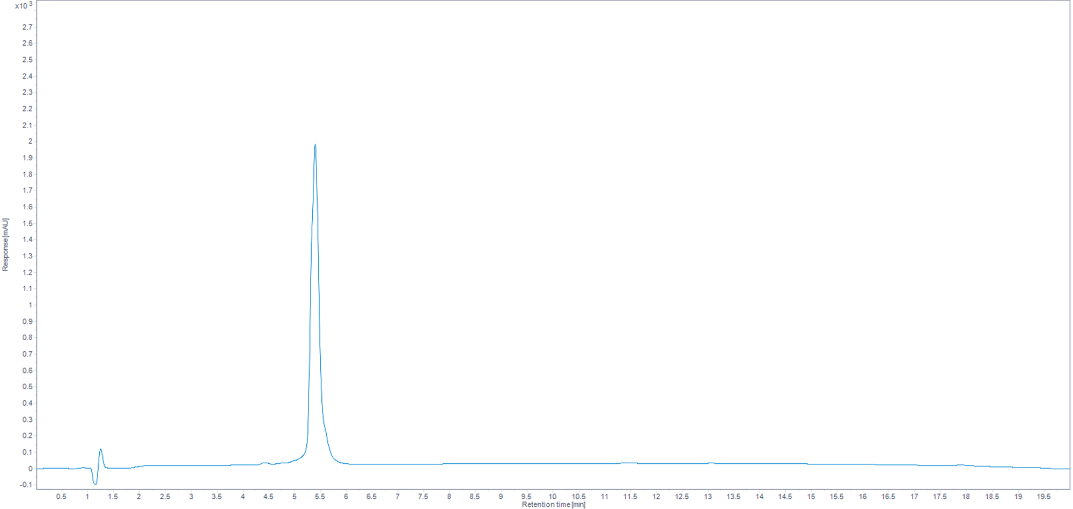


Figure S4. Chromatogram of the analytical HPLC of the purified DOTA-AP-2. The Retention Time of the pure DOTA-AP-2 was measured at 5.39 min.
